# A framework for correcting brain retraction based on an eXtended Finite Element Method using a laser range scanner

**DOI:** 10.1007/s11548-013-0958-8

**Published:** 2013-11-30

**Authors:** Ping Li, Weiwei Wang, Zhijian Song, Yong An, Chenxi Zhang

**Affiliations:** 1 Digital Medical Research Center, Fudan University, Shanghai , 200032 People’s Republic of China; 2 Shanghai Key Laboratory of Medical Imaging Computing and Computer Assisted Intervention, Shanghai , 200032 People’s Republic of China; 3 Shanghai Medical Instrumentation College, Shanghai , 200093 People’s Republic of China

**Keywords:** Brain retraction, Extended finite element method, Laser range scanner, Image-guided neurosurgery system

## Abstract

**Background:**

Brain retraction causes great distortion that limits the accuracy of an image-guided neurosurgery system that uses preoperative images. Therefore, brain retraction correction is an important intraoperative clinical application.

**Methods:**

We used a linear elastic biomechanical model, which deforms based on the eXtended Finite Element Method (XFEM) within a framework for brain retraction correction. In particular, a laser range scanner was introduced to obtain a surface point cloud of the exposed surgical field including retractors inserted into the brain. A brain retraction surface tracking algorithm converted these point clouds into boundary conditions applied to XFEM modeling that drive brain deformation. To test the framework, we performed a brain phantom experiment involving the retraction of tissue. Pairs of the modified Hausdorff distance between Canny edges extracted from model-updated images, pre-retraction, and post-retraction CT images were compared to evaluate the morphological alignment of our framework. Furthermore, the measured displacements of beads embedded in the brain phantom and the predicted ones were compared to evaluate numerical performance.

**Results:**

The modified Hausdorff distance of 19 pairs of images decreased from 1.10 to 0.76 mm. The forecast error of 23 stainless steel beads in the phantom was between 0 and 1.73 mm (mean 1.19 mm). The correction accuracy varied between 52.8 and 100 % (mean 81.4 %).

**Conclusions:**

The results demonstrate that the brain retraction compensation can be incorporated intraoperatively into the model-updating process in image-guided neurosurgery systems.

## Introduction

Image-guided neurosurgery systems (IGNS) are increasingly being used in the operating room (OR) and have been shown to improve surgical visualization and navigation as well as reduce the postoperative tumor residual disease [1–3]. Brain deformation may cause large inconsistencies between images and real anatomy, which is a major source of the error in IGNS [3–5] based on preoperative images. There are three types of brain deformations classified according to their causes. The first type is called brain shift, which is the initial tissue response to changes in the physical or chemical environment inside the skull after opening the dura mater, such as a decrease in intracranial pressure and loss of cerebrospinal fluid (CSF). The second type is retraction, which happens when neurosurgeons use retractors to stretch brain tissue to establish a surgical approach [6, 7]. When a resection is performed by a neurosurgeon, neighboring areas may collapse as a result of gravity or decreased local intracranial pressure. So far, most studies to compensate for brain deformation focused on the first type [3, 5, 8–19], in which the topology of the brain does not change. However, retraction and resection involve topological discontinuity, which have been proven to be more challenging to correct. Thus, related studies have been limited. Generally speaking, the above two latter types of deformation followed by surgical operation usually bring a larger error to IGNS than the first type, and even make navigation totally unreliable. In addition, it is a prerequisite of brain resection to first correct brain retraction because it facilitates tumor access and removal. Therefore, it is of great importance for positional accuracy of IGNS to correct brain retraction.

Most strategies to compensate for intraoperative brain shift fall into two categories: active intraoperative imaging and preoperative image updating based upon the estimated displacements derived from biomechanical models. The use of intraoperative imaging devices, such as intraoperative MR (iMR) [19, 20] and intraoperative ultrasound [11, 15], has been shown to be somewhat costly, cumbersome, and unnecessarily time-consuming [21]. In contrast, alternative strategies have been developed that employ biomechanical models based upon the finite element method (FEM) to warp the preoperative images to reflect the brain shift. This technique takes advantage of limited intraoperative information to create deformed images to compensate for brain shift at a much lower cost than the approaches involving intraoperative imaging. With regard to brain retraction, the problem of using FEM is how to deal with tissue discontinuity. Ferrant et al. [22] used a surface matching algorithm to find the retraction path from intraoperative iMR images, along which the elements touched by the blades of a retractor were deleted, and then updated the preoperative images. Miga et al. [13] proposed a linear poroelastic model based on a consolidation theory and represented the tissue discontinuity by splitting the mesh along existing nonconformal boundary edges of the intraoperative iMR images to obtain the best jagged topological discontinuity. The porcine experiments by their team [23–25] confirmed that this model could recapture 66 % of average tissue motion and reduce the maximum registration error by over 80 %. Sun et al. [26] also employed a linear poroelastic model to correct brain retraction. They tracked the retractors with two charge coupled device (CCD) cameras mounted on an operating microscope, and then used an iterative closest point algorithm to acquire the displacement of the retractor. This model captured approximately 75 % of the cortical motion during tissue retraction.

Actually, FEM cannot handle discontinuities directly and requires one to realign the discontinuity with element boundaries based on mesh adaptation or a remeshing technique; the topology of an initial mesh will be changed into a new mesh that is conformed to the discontinuity [7, 13, 17, 26]. On the contrary, the eXtended Finite Element Method (XFEM) [27] reverses this situation by adding a crack to deal with discontinuity. It generates a mesh without taking the crack into account, and then based on the precise geometry of the crack; it adds extra degrees of freedom (DOFs) to crack-related nodes to handle a crack, or discontinuity. In this way, arbitrarily shaped cracks can be modeled without any remeshing or mesh adaptation. Vigneron et al.  [6, 7, 28, 29] first applied XFEM to a linear elastic model for the correction of brain retraction. They registered intraoperative images from iMR to preoperative images with the application of a nonrigid image registration algorithm to gain boundary conditions (BCs). Although their results showed that the modified Hausdorff distance from the edges of model-updated images to images acquired by iMR was decreased, they only gave a qualitative evaluation. Moreover, iMR scanning is still required to calculate BCs, so this approach may unnecessarily prolong operation time and create extra expenses for patients, which makes it unlikely to become widely available in clinical applications.

In the aforementioned biomechanical model-based strategies, cortical surface deformations for model-updated BCs can be obtained by an intraoperative stereo vision (iSV) system [26], a laser range scanner (LRS) [30–32], or an iMR system [22, 33]. The iSV system needs two CCD cameras attached to the binocular optics of the operating microscope with approximately 1-mm precision. The method of the iMR system requires additional MR scanning after brain retraction. On the other hand, the LRS has been shown to be a portable, easy-to-use, and cost-effective tool for tracking brain retraction surfaces, especially for the inner sub-surface of retractors inserted into the brain.

In this paper, we presented a new framework for the compensation of retraction. A linear elastic biomechanical model is built based on XFEM for an accurate representation of the discontinuity. We utilized a LRS to capture sparse surface point clouds of the surgical field including the outer part and inner sub-surface of retractors inserted into the brain. An innovative surface-tracking algorithm was then applied to register the clouds of the retractors pre- and post-retraction to calculate the displacement of the brain tissue directly contacting with the retractors, which is used as estimated BCs applied to XFEM modeling for driving its deformation. A phantom experiment has shown that the framework we presented was feasible.

## Materials and methods

### Brain retraction correction framework

**Fig. 1:**
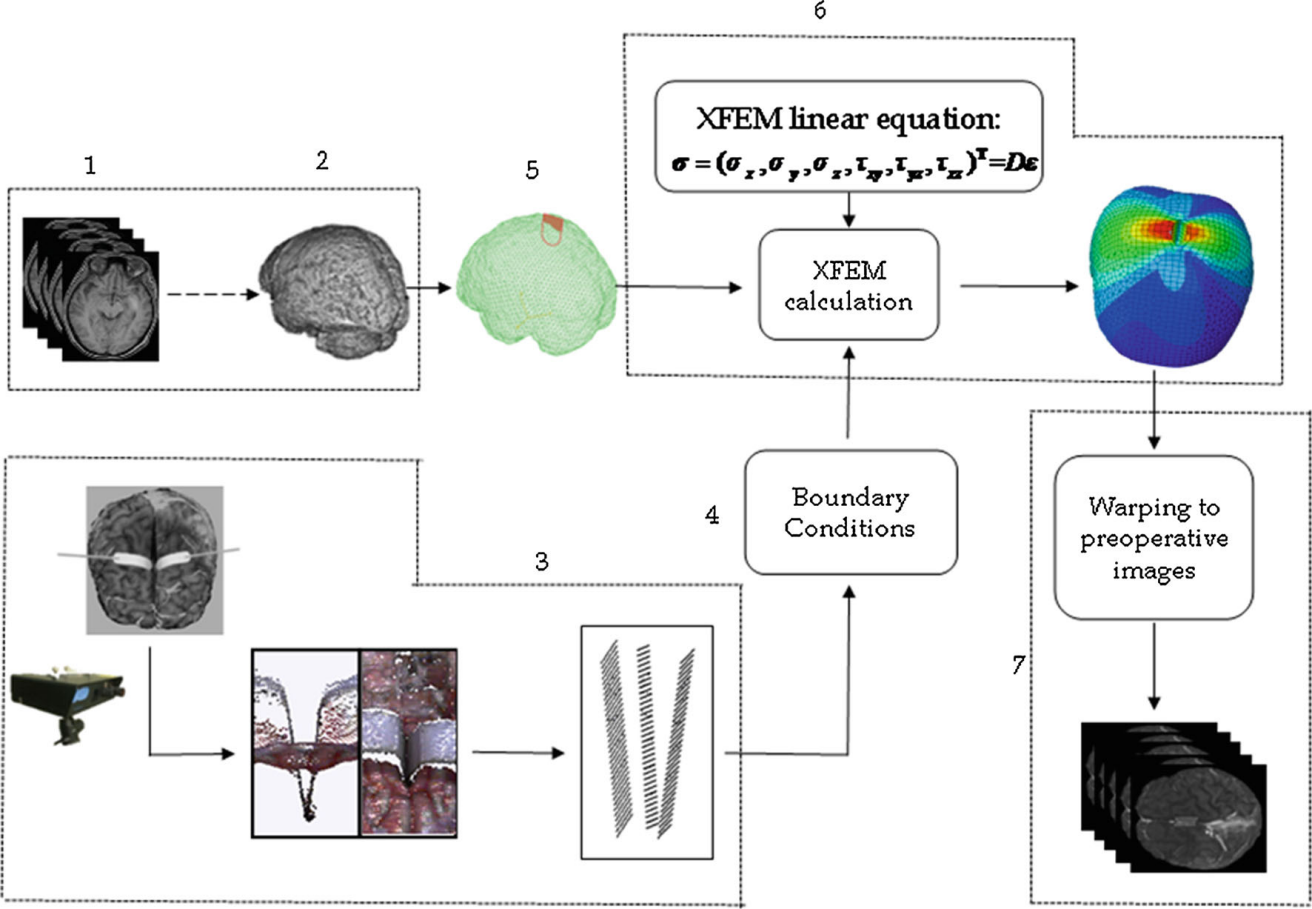
An XFEM-based framework was used to correct brain retraction using a LRS. Steps *1*–*2* are done before the operation. All the intra-operation procedures begin from Step *3* when the skull was opened, the dura mater was removed, and the retractors were inserted into the brain

As shown in Fig. 1, the numbers represent the procedure steps. In Steps 1 and 2, the craniotomy is performed, followed by a brain shift caused by gravitational sag of brain tissue in response to the craniotomy; the brain shift is assumed to have been well estimated and is described in [14]. In Step 2, these images are treated as pre-retraction images from which the brain tissue can be segmented first in coarse segmentation and later in fine segmentation. Then, the images are generated into a uniform hexahedral volumetric mesh. In Step 3, a brain retraction surface-tracking algorithm is used to acquire the surface displacement of the brain tissue when two retractors are inserted and stretched out. Step 4 is the acquisition of BCs; one of the BCs is the surface displacement of brain tissue, which is obtained in Step 3, and the other is the zero displacement area of brain tissue. In Step 5, a linear elastic XFEM model is built. With BCs and the biomechanical model available, Step 6 is used to initialize and solve the XFEM equations to obtain the updated mesh, which is used to warp the pre-retraction brain images by a modified back-interpolation algorithm in Step 7 resulting in the model-updated images. Details of Steps 3–7 will be described in the following subsections.

### Brain retraction surface-tracking algorithm

This brain retraction surface-tracking algorithm is used to acquire surface movement of brain tissue in the operating field intra-operatively after two retractors are inserted. The acquired surface movement will be used as BCs to drive model updating. The point clouds representing the post-retraction surface of the retractors need to be registered to those of pre-retraction so that the displacement of brain nodes directly in contact with the retractors can be calculated.

#### Acquisition of retractor point cloud

**Fig. 2:**
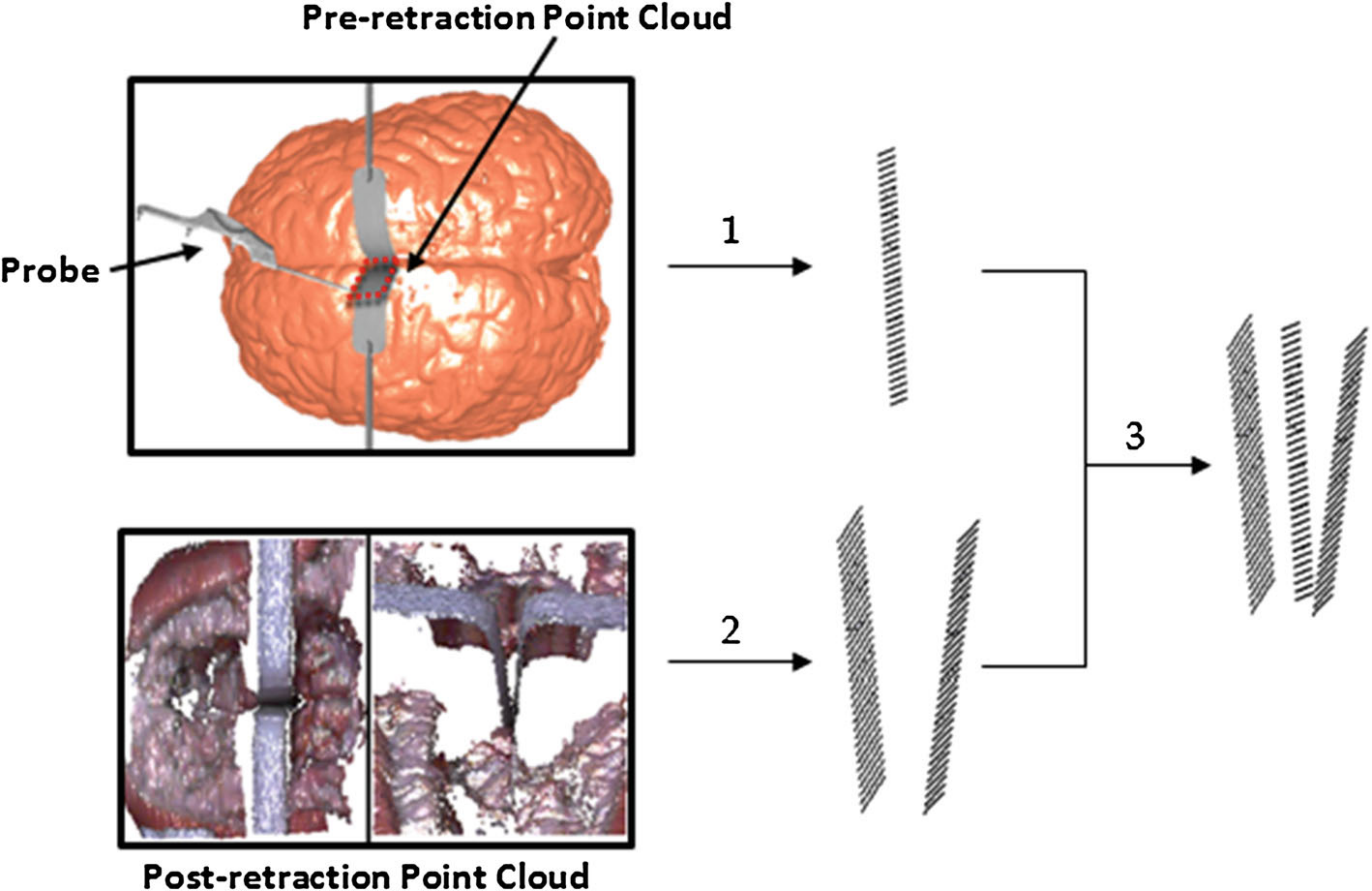
Acquisition of retractor point clouds before and after the retractors are inserted. In Step *1*, the pre-retraction point cloud can be tracked by a navigation probe. In Step *2*, the inner V-shaped sub-surfaces of retractors inserted into the brain tissue were obtained by LRS scanning. In Step *3*, both pre- and post-retractor point clouds are transformed into the same image space

A plane describing the position and orientation of retractors and inter-hemispheric fissure was determined by a navigation probe [14]. The retractors were first inserted into the brain tissue, vertical to the floor, and parallel to the direction of falx cerebrum. Before the retraction, the probe was used to capture the coordinates of specific parts of the retractors, as shown in Step 1 of Fig. 2. The pre-retraction point clouds of retractors were then constructed by using a combination of the coordinates and the dimensions of the retractors. These coordinates could assist us in quickly locating the pre-retraction point clouds of the retractors. With the aid of the point clouds of retractors, elements that were cut by the retractors and all related nodes were identified and then used to initiate XFEM calculation.

During the brain retraction, the brain surface point clouds including the inner V-shaped sub-surfaces of retractors can be acquired at any time by scanning the exposed surgical area of the tissue retraction using the LRS. Sometimes, LRS cannot capture the whole geometric shapes of the retractors because of partial obstruction of the laser. The point cloud model of the retractors prepared beforehand can be rigidly registered to the part captured by the LRS to augment the part, as shown in Step 2 of Fig. 2.

The pre-retraction point clouds were transformed by transformation matrix $T_{\mathrm{Ref}\text {-}\mathrm{Img}}$ [14] from the reference frame space to pre-retraction image space. The post-retraction point clouds captured intra-operatively by LRS were also converted from the LRS space to the pre-retraction image space by transformation matrix $T_{\mathrm{LRS}\text {-}\mathrm{Img}}$ [14]. These two groups of point clouds of the retractors were incorporated in the same image space and can be used to calculate the displacement of retractors, as shown in Step 3 of Fig. 2.

### Point cloud registration

To calculate the displacement of retractors, a coherent point drift (CPD) algorithm [34] was utilized to nonrigidly register the post-retraction point clouds of retractors to the pre-retraction ones. Considering the density of two point clouds, the denser pre-retraction point clouds of retractors represented Gaussian mixture model (GMM), while the sparser post-retraction point clouds acquired by LRS represented sample data points of the denser point clouds. Hence, the problem of point set registration was successfully transformed into the problem of the Gaussian mixed density estimation by estimating the probability distribution of the denser pre-retraction point clouds on the basis of the ones of the sparser post-retraction point clouds.

Based on the registration results, displacements of the inner sub-surfaces of the retractors can be obtained. However, the displacements cannot be directly imposed on the XFEM-based biomechanical model and should be moved a distance equal to the thickness of the retractor in the direction of retractor surface norm. Then, the displacements of nodes in direct contact with the retractors were obtained and will later be used in the brain biomechanical model as part of the BCs.

### Linear elastic model and solution of the XFEM equation

A linear elastic model is so far the simplest model and yet sufficient for brain deformation estimation [8]. It treats the brain as an isotropic tissue and defines it with a series of partial differential equations. These differential equations are mathematically solved by the following linear equation [14]:

1 $$\begin{aligned} { Ka}=P \end{aligned}$$

where $P$ is the force vector, $a$ is a nodal DOF vector, and $K$ is an XFEM stiffness matrix characterizing the properties of the material. Matrix $K$ is determined by two independent parameters: Young’s modulus (E), which relates a material’s response to tension or stretching, and Poisson’s ratio ($\nu$), which is the ratio of lateral contraction due to longitudinal stretch.

Theoretically, FEM cannot handle discontinuity because nodal shape functions (NSFs), which determines the biomechanical properties of nodes, are continuous functions [35]. XFEM improves FEM and adds extra DOFs to nodes that are related to discontinuity. This improvement makes mesh adaptation [36–38] or remeshing [39, 40] unnecessary [41]. The XFEM displacement field is displayed as follows:

2 $$\begin{aligned} u^{\mathrm{XFEM}}(x)&= \sum _{i\in I} {\varphi _i (x)u_i } +\sum _{j\in J} {\varphi _j (x)H_j (x)a_j }\nonumber \\&\quad +\sum _{m\in M} {\varphi _M (x)\left( {\sum \limits _{l=1}^4 {F_l (x)} C_k^l } \right) } \end{aligned}$$

where $u^{\mathrm{XFEM}}$ is XFEM displacement. The first term on the right-hand side represents the FEM displacement in which $I$ is the set of FEM nodes, $\varphi _i$ are the FEM NSFs, and $u_i$ are the FEM DOFs. Additional DOFs $a_j$ and $c_k^l$ are added to set $J$ and $M$ to define discontinuity for XFEM. The set $I$ is composed of both nodes in set $J$and set $M$ as follows:

3 $$\begin{aligned} J,M\in I,J\cap M=\phi \end{aligned}$$

**Fig. 3:**
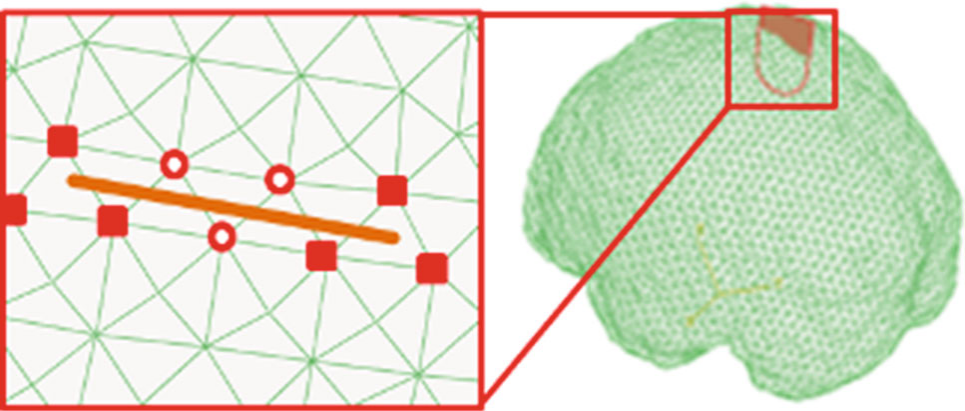
The schematic of XFEM. The nodes are shown as the *red hollow circle* when elements are completely cut by the crack. When elements are completely cut by the crack, all related nodes are shown as the *red solid square*

When elements are completely cut by the crack, all related nodes shown as the red hollow circle in Fig. 3 form the set $J$. To represent the discontinuous characteristics of set $J$, Heaviside step functions are added in the second term, which are as follows:

4 $$\begin{aligned} H(x)=\left\{ {{\begin{array}{ll} {1,(x-x^{*})\cdot e_n \ge 0} \\ {-1,(x-x^{*})\cdot e_n \le 0} \end{array} }} \right. \end{aligned}$$

where $$x$$ is the position of a point in set $$J,\,x^* $$ is the position of the point on the crack that is closest to $$x,\,e_n$$ is the outward normal to the crack at $$x^* $$.

When elements are partially cut by the crack, the related nodes shown as the red solid square in Fig. 3 form set *M*. In the third term of Eq. (2), to represent the discontinuous characteristics of $M,\,F_l$ combine the radial and angular behavior of the asymptotic linear elastic crack tip displacement, which are as follows:

5 $$\begin{aligned} \left\{ {F_l (r,\theta )} \right\}&= \left\{ \sqrt{r}\sin \frac{\theta }{2},\sqrt{r}\cos \frac{\theta }{2},\quad \sqrt{r}\sin \frac{\theta }{2}\sin \theta ,\sqrt{r}\cos \frac{\theta }{2}\sin \theta \right\} \end{aligned}$$

where $r$ and $\theta$ are the normal and direction vectors of the position of a point $x$ of set $M$, respectively.

### Acquisition of BCs

To achieve a unique solution to the XFEM equations (1), two types of BCs need to be determined and imposed. One is the zero displacement BC, which is traction and stress free. The zero displacement area of brain tissue is described as the location of the brain stem and manually selected by experienced doctors. The other type of BC is the displacement of certain crack-related nodes in the operating field where two retractors are inserted into brain issue. This BC can be calculated by the brain retraction surface-tracking algorithm. These two types of BCs are imposed to the XFEM-based linear elastic model to achieve a unique solution and updated mesh, from which the displacement of all nodes and crack-related information can be obtained.

### Biomechanical model updating

The updated mesh will be exploited by the modified back-interpolation algorithm, which is not only able to warp the pre-retraction images and use ray casting to visualize them [41, 42], but also able to recognize the crack generated by brain retraction. Regarding crack representation, our modified algorithm can discriminate two level sets [41, 42]. One level set $\psi$ represents the distance from the position of a node to the position of a point on the crack that is closest to this node. The other level set $\phi$ represents the distance from the position of a node to the position of a point on the crack tip. The representation of a crack includes two level sets only when the crack ends within the node support. This algorithm translated them into a shape of discontinuity in the updated images.

### Evaluation

**Fig. 4:**
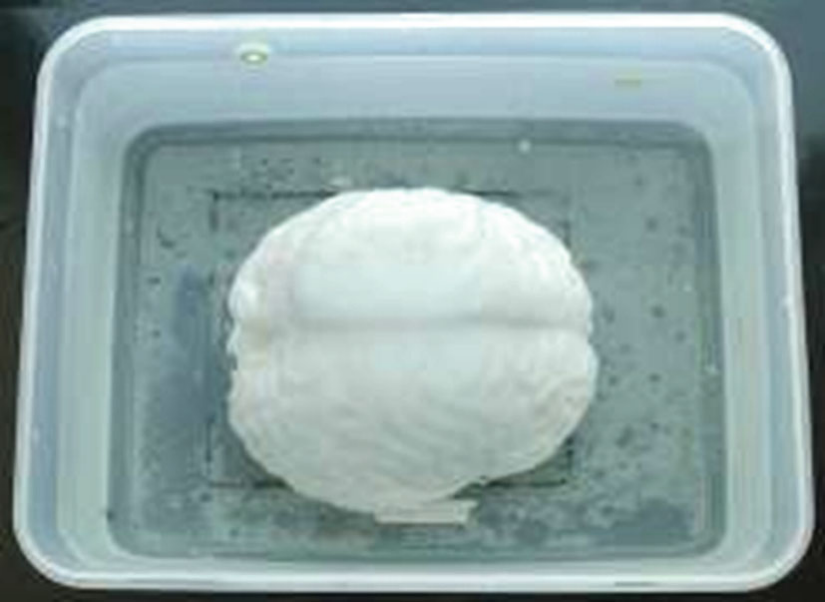
The brain phantom was fixed in one Plexiglas box with five fiducial landmarks. It has a similar shape to the human brain and similar linear elastic parameters of Young’s elasticity modulus and Poisson’s ratio as the human brain to ensure that the phantom has the similar elastic behavior as the brain

A phantom experiment similar to [31, 43] was conducted to quantify the fidelity of our XFEM-based framework and to quantitatively validate retractor-induced brain deformation. One brain phantom (1.5 kg) shown in Fig. 4 was made in the University of Science and Technology Beijing (USTB).

PVA-C as polymer becomes harder with an increase in the number of freeze–thaw cycles. It is manifold, notable for building biological tissue-mimicking phantoms [44]. Previous PVA-C phantom studies have employed 6 % by weight PVA and have yielded Young’s modulus values ranging from 2.5 to 5.4 kPa through changing of the number of freeze-thaw cycles [43]. Referencing on the similar method [43], the 6 % solution of PVA with three freeze–thaw cycles was used to construct brain phantom. The brain phantom achieved Young’s modulus value of 3 kPa, which is similar to elasticity parameters of the actual brain tissue [14, 17, 18]. Therefore, we use Young’s modulus value of 3 kPa in the finite element analysis.

It is hard to seek to obtain Poisson’s ratio of soft PVA-C materials, because soft materials have different deformation behavior where the deforming pattern is irregular and pointwise [45]. In earlier work, authors [46] measured Poisson’s ratio of PVA-C under different experimental conditions and the results indicated the Poisson’s ratio value ranges from 0.42 to 0.45. We made several experiments using different Poisson’s ratio ranging from 0.42 to 0.45 and found that the results were not affected by different Poisson’s ratio. Thus, Poisson’s ratio of 0.45 was used in XFEM analysis.

This phantom has nearly a linear stress–strain curve within deformation of $<$20 %, so it can model an isotropic linear elastic material, which is characterized by two parameters: Young’s elasticity modulus and Poisson’s ratio [35]. During the experiment, this phantom was fixed on its bottom face in one Plexiglas box with five fiducial landmarks and deformed with a uniform force distributed along the super midline on the top face, resulting in the geometric coincidence of fissure and retractors. The retractors were inserted in the regions of the frontal and parietal lobe. To detect tissue retraction, 23 stainless steel beads (1.5 mm in diameter) were embedded into the phantom. 4, 12 and 7 beads were placed in the frontal, parietal, and occipital lobes, respectively. Twenty-one of the beads were placed in the shallow brain tissue except that both 22nd and 23rd beads were in deep brain tissue. These embedded beads moved together with the brain tissue, and they worked as the sampling points in the brain tissue when measuring the displacement from retraction. CT images after retraction of the phantom (using a Siemens SOMATOM Definition AS, spiral mode) and the beads in these images could then serve as a basis of comparison with the model-updated images acquired from our framework. These images were reconstructed into a spatial resolution of $512 \times 512 \times 421\,\hbox {mm}^{3}$ with a voxel size of $1 \times 1 \times 1\,\hbox {mm}^{3}$. A 14-mm-wide Plexiglas retractor blade simulating NA20010 (JZ Surgical Instruments, Shanghai, China) was used to stretch out the brain phantom.

Model-updated images and the post-retraction CT images could be registered into the same pre-retraction image space in the IGNS system and compared together to evaluate the accuracy of our framework. We used three quantitative criteria. One was to calculate the forecast error defined in formula (6), which is the Euclidean distance between the positions of corresponding beads separately in these two groups of images; the second was the correction accuracy defined in Eq. (7).

6 $$\begin{aligned} \hbox {Forecast Error}=\left\| {C_{\mathrm{model}\text {-}\mathrm{updated}} -C_{\mathrm{post}\text {-}\mathrm{retraction}} } \right\| _2 \end{aligned}$$

7 $$\begin{aligned} \hbox {Correction Accuracy}&= \left( 1-{\left\| {C_{\mathrm{model}-\mathrm{updated}} -C_{\mathrm{pre}\text {-}\mathrm{retraction}} } \right\| _2 }\right. \nonumber \\&\quad \left. /{\left\| {C_{\mathrm{post}\text {-}\mathrm{retraction}} -C_{\mathrm{pre}\text {-}\mathrm{retraction}}} \right\| _2 } \right) \nonumber \\&\quad \times \, 100\,\% \end{aligned}$$

where $C_{\mathrm{pre}\text {-}\mathrm{retraction}} , C_{\mathrm{model}\text {-}\mathrm{updated}}$, and $C_{\mathrm{post}\text {-}\mathrm{retraction}}$ stands for the beads’ coordinates in the pre-retraction, model-updated, and post-retraction CT images, respectively.

The third criterion was used to evaluate the edge alignment of the brain contour by our framework morphologically. Here, the modified Hausdorff distances based on Canny edges extracted from pre-retraction CT, post-retraction CT, and model-updated images were computed. The modified Hausdorff distance [47] ${H}\left( {A,B} \right)$ between two sets of points A and B is defined as:

8 $$\begin{aligned} H\left( {A,B} \right) =\max \left( {h\left( {A,B} \right) ,h\left( {B,A} \right) } \right) \end{aligned}$$

where $h\left( {A,B} \right)$ is the directed Hausdorff distance, which is a measure of the distance from point set A to point set B.

## Results

**Fig. 5:**
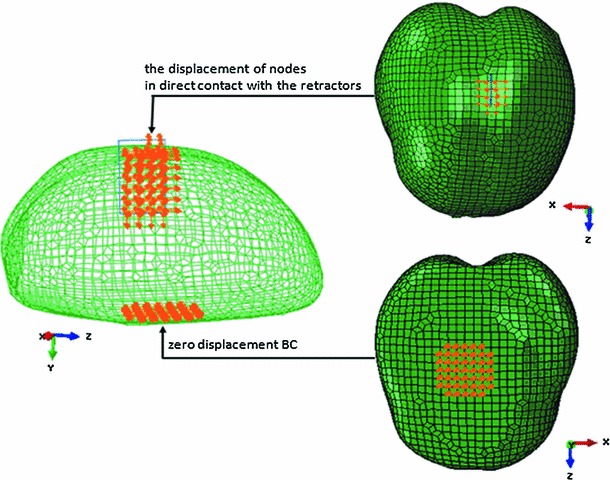
Graphic illustration of BCs. The *orange*, higher dense section of points indicates two types of BCs imposed to the XFEM-based linear elastic model

Figure 5 is a pictorial representation of the distribution of BCs for a volume mesh description of the phantom. It illustrates various zones within the model that support different boundary data. In the region of the craniotomy in the modeled fissure, the displacements of surface in contact with the retractor are calculated by the brain retraction surface-tracking algorithm. In the region of the bottom area of the phantom, traction-free conditions have been prescribed with no drainage.

**Fig. 6:**
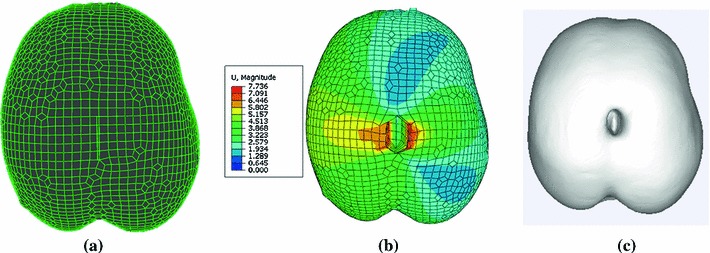
The workflow of our XFEM brain retraction correction framework. **a** Uniform pre-retraction hexahedral mesh (10,675 nodes and 9,316 elements) with retractors on the surgical path. **b** Updated mesh with color coding corresponding to different magnitudes of displacement (*red indicates* the maximum, *blue indicates* the minimum). **c** Three-dimensional surface rendering of updated images by ray-casting algorithm

Two retractors were inserted into the brain phantom and stretched separately to the right with a maximum distance of 7.0 mm and to the left with a maximum distance of 6.3 mm, respectively. Figure 6 briefly illustrates the workflow of our XFEM-based framework in three dimensions. Figure 6b shows that the maximum displacement is found just around the crack.

**Fig. 7:**
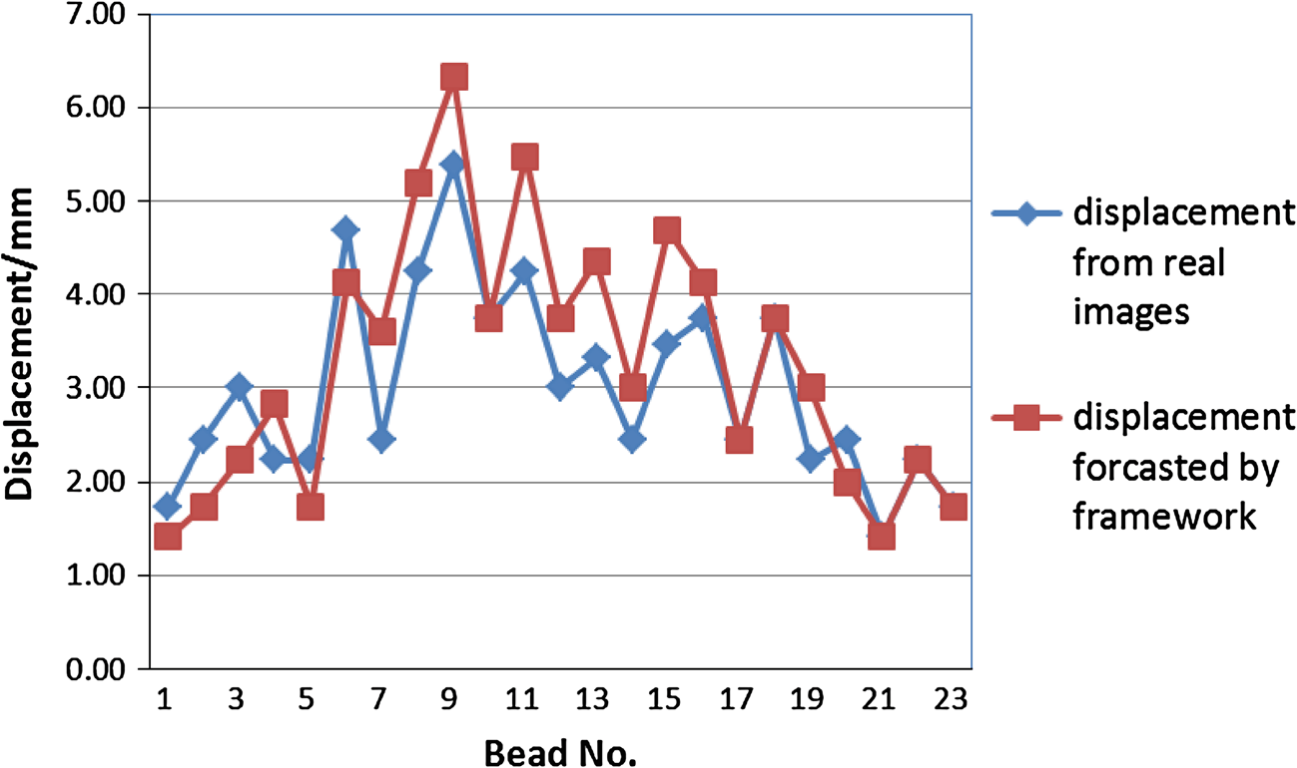
A comparison between the actual displacements of 23 beads implanted into the brain phantom (from post-retraction CT images) and the predicted ones (from the XFEM-based framework). The *red square* indicates predicted displacements using our framework. The *blue diamond* indicates the actual deformation displacements

Figure 7 illustrates the displacements of the 23 beads implanted into the brain phantom from the updated images and post-retraction CT images. It indicates that the XFEM-based brain deformation displacement ranges from 1.41 to 6.32 mm, and the actual deformation displacement is from 1.41 to 5.39 mm. The trend of these two displacement curves is the same, and the red curve (our framework) can predict the retraction deformation well.

**Fig. 8:**
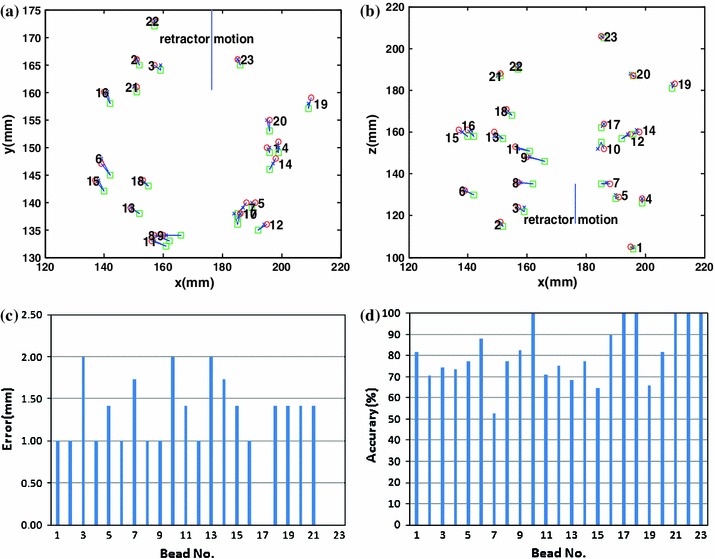
Results of 23 pre-retraction, measured and computed bead locations presented in orthogonal views. **a** Coronal view (*X*–*Y* plane) comparison between pre-retraction and measured or calculated bead trajectories. *Square* preoperative, *x* measured, *o* calculated. **b** Axial view (*X*–*Z* plane) comparison between pre-retraction and measured or calculated bead trajectories. *Square* pre-retraction, *x* measured, *o* calculated. The initial retractor position is represented by the *solid line* in each plane and the direction of retraction is shown in (**a**) and (**b**). **c** The forecast error. **d** Correction accuracy

Figure 8 shows comparisons of bead locations in the pre-retraction CT images, measured from the post-retraction CT images and calculated by our framework in orthogonal views. It gives a detailed pictorial analysis about our framework’s forecast ability and accuracy. Figure 8a indicates a coronal view (*X*–*Y* plane) comparison between pre-retraction and measured or calculated bead locations. Figure 8b compares bead displacements from pre-retraction to measured and calculated in the axial view (*X*–*Z* plane). From Fig. 8a, b; in the left side of the retractors, beads calculated tend to move to the left and top, while in the right side of the retractors, beads calculated tend to move to the right and bottom. The forecast error is not focused on specific frontal, parietal and occipital lobe regions. Figure 8c shows that the forecast error varies between 0.0 and 2.0 mm (mean 1.19 mm). The forecast errors of the 18th, 22nd, and 23rd beads which are far from the retractors and located in deep brain tissue are zeros. Figure 8d illustrates that the correction accuracy between the forecasted and actual results varies between 52.8 and 100 % (mean 81.4 %). From Fig. 8c, d, the forecast errors have no connection with the regions where the beads were embedded.

**Fig. 9:**
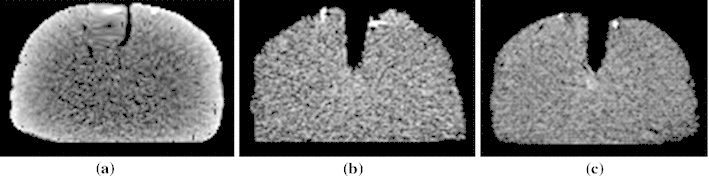
Warped images updated by **a** the traditional back-interpolation algorithm and **b** the modified back-interpolation algorithm. **c** Intraoperative images acquired by CT

When warping pre-retraction images, the modified back-interpolation algorithm could identify both nodal displacements and crack-related information. Figure 9 illustrates the differences between warped images by the traditional back-interpolation (see Fig. 9a) and the modified back-interpolation algorithm (see Fig. 9b). Figure 9c illustrates the intraoperative images acquired by CT. The crack shapes of Fig. 9a, c are obviously different, while those in Fig. 9b, c are similar.

**Fig. 10:**
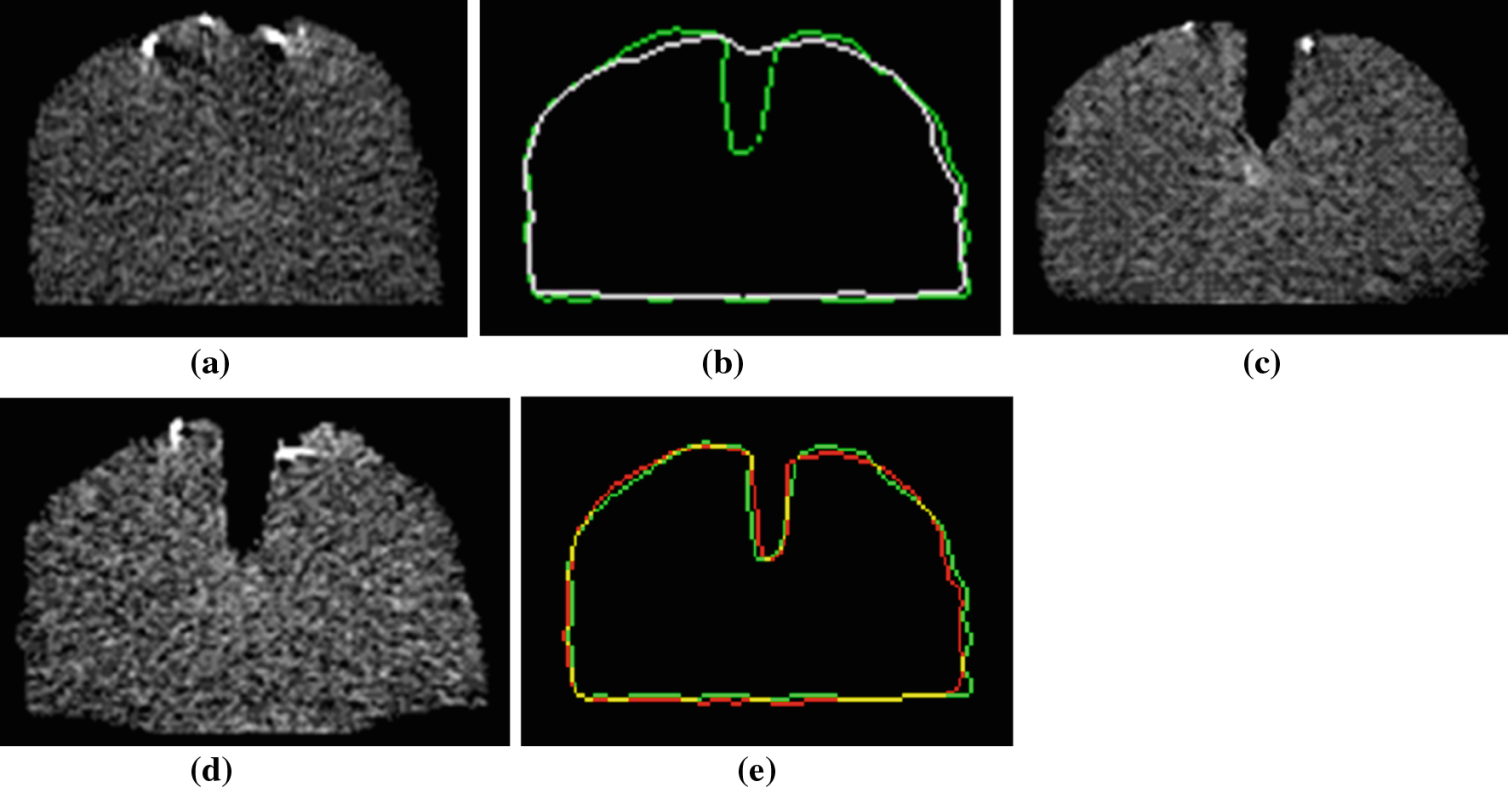
A comparison of a phantom contour in one pre-retraction CT image, post-retraction CT image, and updated image. **a** One slice of pre-retraction CT image. **b** Combination of the contour of the pre-retraction CT slice (*in white*) with the corresponding one of the post-retraction CT image (*in green*). **c** One slice of actual post-retraction CT image. **d** The updated image of the same slice using our framework. **e** Juxtaposition of contours of the same slice from the updated image (*in red*) and that from the post-retraction CT image (*in green*). The *yellow contour* represents position where the *red* and *green contours* coincide with each other

The modified Hausdorff distance decreased from 1.1 mm for the set of edges extracted from 19 pre-retraction CT images to 0.8 mm for the set of edges extracted from 19 updated images using our framework. Figure 10a shows one slice of pre-retraction CT images. Figure 10c shows one slice of an actual post-retraction CT image. Figure 10b combines a phantom contour from a pre-retraction CT image with the one from a post-retraction CT image. Figure 10d displays one slice of the updated images using our framework. Figure 10e juxtaposes the phantom contour from the updated image and the actual brain retraction CT image in Fig. 10c. In Fig. 10b, e, the updated images by our XFEM-based framework were in accordance with the actual CT images.

## Discussion

Brain retraction usually brings a large distortion to IGNS or even makes the navigation system totally unreliable. Most studies employed FEM-based biomechanical models to warp preoperative images to reflect brain retraction. However, it is difficult for FEM to deal with tissue discontinuity, while XFEM may be a good alternative. BCs at the retractor blade surface can also be improved by introducing a LRS-based method. These advances bode well for the model and its ability to capture tissue deformation from complicated surgical procedures such as retraction. A validation experiment of tissue retraction has been completed using a brain phantom. Detailed measurements of tissue motion were compared with model calculations. Certainly, the 81.4 % motion recapture rate found in our phantom experiment would constitute a significant improvement over not using any form of tissue motion compensation in the OR.

Our linear elastic biomechanical model was based on XFEM, which adds extra DOFs to nodes related to discontinuity. This improvement compared with FEM makes mesh adaptation or remeshing unnecessary. Its first application by Vigneron et al. [6, 7] has shown great potential to successfully correct brain retraction and brain resection for IGNS. In our framework, this novel method combined with the LRS-based surface-tracking system could quickly identify the information about brain retraction. It demonstrated an improved accuracy without engaging intraoperative imaging facilities.

In the work presented here, the application of LRS in neurosurgery can track sufficient surface points of the retractors especially for the inner sub-surface of retractors inserted into the brain and measure intraoperative brain retraction surfaces in an automatic and rapid fashion instead of using iMR images. Compared with serial iMR scanning, serial LRS scanning did not prolong operation time or change operation schedules. LRS is easy-to-use, cost-effective, and easy to operate for tracking brain retraction surfaces. Furthermore, the LRS unit could readily capture the surface points of the retractors within the surgical field in the OR.

The accuracy of the BCs directly influenced the final accuracy and error of the framework. From Fig. 5, two distinct displacement trajectories could be easily identified. From Fig. 8a, b, the calculated locations of beads in the left side tend to be left and top, while those in the right side tend to be right and bottom. This phenomenon, no doubt, rose by the slight distortions of the BCs. Moreover, the locations of beads and voxel size of framework also took effect. In this framework, two types of BCs were employed; one was zero displacement BCs, and the other was the displacement of certain directly crack-related nodes. The center of two types almost lies in the same axes (*X* axes in Fig. 5). Therefore, tissue along the *Z* axes, from top to bottom of the phantom, moved from maximum to zero displacement. Because the accuracy of our biomechanical model heavily depended on the accuracy of BCs, tissue that was near the bottom far away from the crack moved only slightly, e.g., 0.5 mm. However, because of the voxel size, the displacement captured by our framework was artificially increased to 1.00 mm. The misalignment of beads embedded in this area caused the accuracy in our framework to decrease. Meanwhile the operator should use the probe carefully when capturing the plane describing initial position and orientation of the retractor to reduce the operative error in the neurosurgical procedures.

Because the embedded beads moved together with the brain tissue, we could numerically evaluate the effectiveness of our framework through the displacement of these beads. Unlike Platenik et al. [24, 25], implanting beads only near the inter-hemispheric fissure, our stainless steel beads were embedded in a wide area including the frontal, parietal, and occipital lobes. In addition, compared with previous in vivo or in vitro experiments, more beads were used here. Based on the coordinates of the beads, the forecast error indicated that the coordinates of the same bead calculated by our framework agree well with the ones measured from actual images. The closer the distance was between two locations of the same bead, the better accuracy was achieved by the framework. However, this was not sufficient. The forecast error did not take pre-retraction beads into account. The correction accuracy could distinctively represent the percentage of the displacements of beads modeled by our framework to the real situations.

Morphological evaluation is also critical for the evaluation of our framework, so edge detection should be a better choice. The modified Hausdorff distance method measured morphological consistency based on the visual comparison of slice contours. Similarly to Vigneron et al. [6, 7], the edges between model-updated images and post-retraction images looked more like each other by their appearance. Moreover, the numerical results of modified Hausdorff distances were much smaller, which meant quantitatively that the numerical distance of these two pairs of edges decreased and our framework showed an improved performance.

We found that the linear elastic model was not appropriate when the framework was used to correct large retraction deformation (large displacement of more than 13 mm in stretched zones near the retractor in surgery). It may be because larger displacement results in larger discontinuity, which is not in accordance with its theoretical foundations. A nonlinear model such as the viscoelastic material model may be more appropriate, and we plan to study on this model in our future research.

We evaluated the XFEM framework by a brain phantom experiment. Although the phantom simplified the evaluation procedures of the experiment, it may be a little different from a human brain retraction case. The phantom experiment has two limitations: (1) the brain is confined to the skull, which restricts the brain retraction deformation to some extent during surgery, but there are no such confinements for the phantom experiment; (2) the brainstem is not considered to move during surgery, whereas the bottom area of the brain phantom fixed to the Plexiglas box has been treated as zero displacement.

**Fig. 11:**
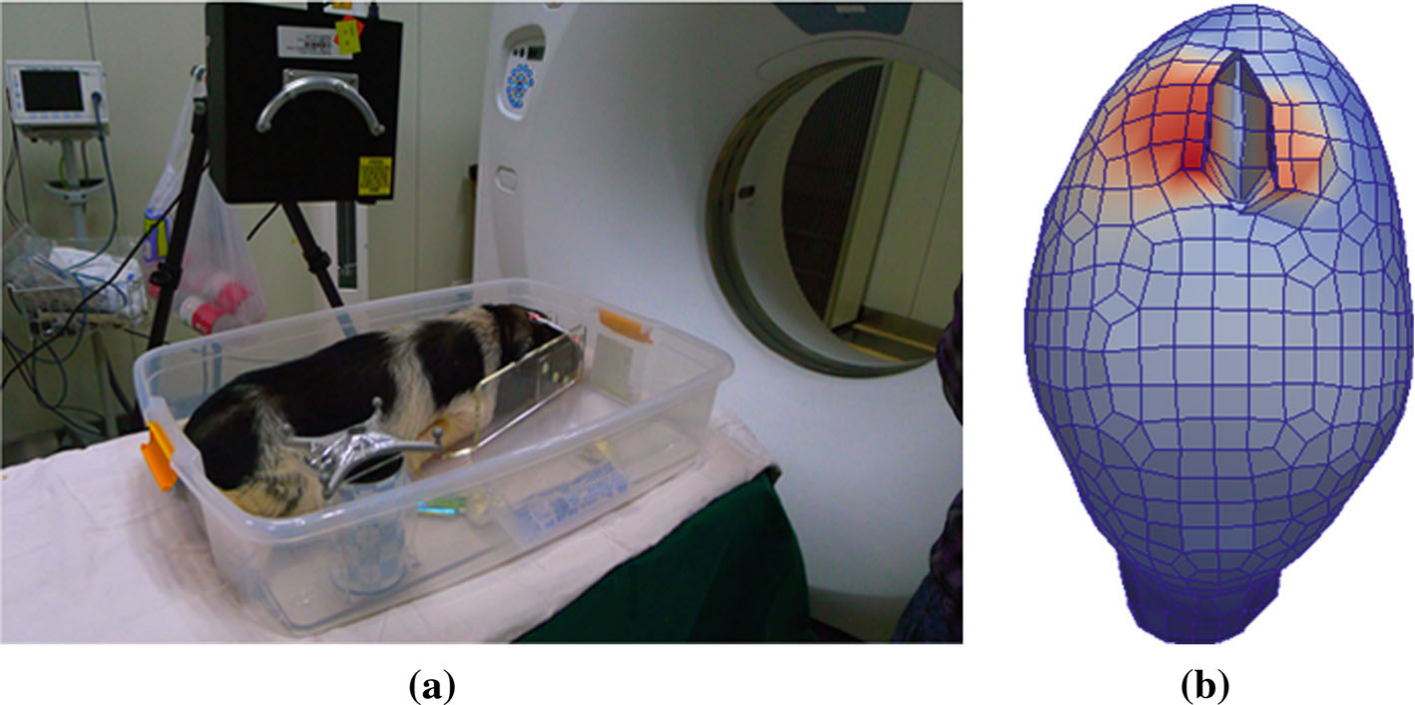
The pig experiment. **a** Pig experiment scene. **b** The updated mesh predicted by our framework

Our aim is to apply this framework to the clinical application for patients. Further verification experiments have been implemented on a few cases of pigs (shown in Fig. 11) after being approved by the Institutional Animal Care and Use Committee. We used intraoperative CT data to evaluate the capability of the framework as a reference standard. The results of pig experiments initially showed that the retraction deformations predicted by the present framework agree well with the ones observed intraoperatively.

In the experiment, CT images used as the gold standard had low resolution for soft tissue; therefore, it degraded our brain segmentation performance. We plan to use iMR scanning in the next experiments, which will improve the segmentation algorithm.
